# Rapid Expansion of a Teledermatology Web Application for Digital Dermatology Assessment Necessitated by the COVID-19 Pandemic: Retrospective Evaluation

**DOI:** 10.2196/36307

**Published:** 2023-07-26

**Authors:** Shareen Muthiah, Fiona E Craig, Siobhan Sinclair, Grant Wylie, Donna Torley, Terence H Wong, Colin A Morton

**Affiliations:** 1 Stirling Community Hospital Stirling United Kingdom; 2 Queen Elizabeth University Hospital Glasgow United Kingdom

**Keywords:** dermatology, asynchronous, outpatient, consultation, teledermatology, telehealth, telemedicine, consultation, digital health, eHealth, digital consultation, dermatologist, skin, return patient, digital assessment, online platform

## Abstract

**Background:**

The COVID-19 pandemic necessitated a change in the provision of outpatient care in dermatology.

**Objective:**

A novel, asynchronous, digital consultation platform was codeveloped with 2 National Health Service dermatology teams to improve access and enhance choice in outpatient care.

**Methods:**

The rollout of the platform was accelerated during the initial COVID-19 lockdown, and its wider use across 2 Scottish health boards was retrospectively evaluated. Integrated with the hospital booking system and electronic patient record, the platform provides an alternative to face-to-face consultations, using information and images submitted by the patients.

**Results:**

In total, 297 new patient consultations and 108 return patient consultations were assessed, and 80% (324/405) of the images submitted were of satisfactory quality. The consultations were, on average, 3 minutes shorter than equivalent face-to-face interactions, and a total of 5758 km of patient travel was avoided. Outcomes included web-based reviews (66/405, 16.3%), face-to-face reviews (190/405, 46.9%), biopsies (46/405, 11.4%), discharge (89/405, 22%), and other treatments or investigations (14/405, 3.5%). High levels of patient satisfaction (92/112, 82.1%) were reported.

**Conclusions:**

Digital dermatology assessments are now included in the choices for consultation types that are available to patients, helping to augment service capacity during pandemic recovery.

## Introduction

Dermatologists have been identified as “behind the curve” in digital innovation [1]. Prior to the pandemic, teledermatology had not been used as a consultation method in outpatient clinics within Scotland. Although lesion photo triage was available in a limited number of health boards, its primary purpose was for enhanced referral triage, and it required patients to have face-to-face contact with a clinical photographer. Pandemic restrictions necessitated a reduction in face-to-face consultations, with priority given to essential cancer and emergency services. To provide continuing patient care, existing outpatient models were redesigned to enable the use of store-and-forward asynchronous teledermatology. In comparison with synchronous real-time teledermatology (primarily video consultations), asynchronous teledermatology involves electronically transmitting clinical details and images to a clinician for review at a different time and location [2]. This method was preferred, as it has been widely reviewed, has the potential to improve clinical efficiency [3-5], provides flexibility and convenience to both clinicians and patients, and confers environmental benefits through reduced travel and carbon emissions [6]. Asynchronous teledermatology also permits the uploading of higher-quality images of specific lesions by patients. This is preferable to relying on lower-resolution videos and avoids the potential pitfalls of slow internet connection speeds, which are issues in the case of synchronous teledermatology [7].

A government-sponsored initiative sought to fund innovative solutions for use in modern health care settings. Dermatology, with its high volumes of outpatient consultations, was identified as an area with development potential, and software companies were asked to pitch their ideas for novel digital systems to improve health care efficiency. Through this, an asynchronous teledermatology platform was developed collaboratively by dermatology teams from 2 Scottish health boards (National Health Service [NHS] Forth Valley and NHS Greater Glasgow and Clyde) and a digital agency [8]. NHS Forth Valley serves a diverse geographical area in the heart of Scotland, and NHS Greater Glasgow and Clyde is the largest NHS organization in Scotland and one of the largest in the United Kingdom. Clinicians’ and patients’ views were taken into account when designing the system to make it user-friendly at both ends of the interface.

The platform allows patients to submit information and photographs of their skin condition, which are then assessed by a clinician. Afterward, the patients receive a response, and a summary of the consultation is sent to primary care and stored on the patients’ electronic records.

Although the platform was originally designed for follow-up consultations, the challenges posed by the COVID-19 pandemic lockdown prompted its more widespread use, including for new consultations. We therefore evaluated its feasibility as a consultation platform, with respect to efficiency, patient satisfaction, and its use as a diagnostic or triage tool.

## Methods

### Overview

**Figure 1:**
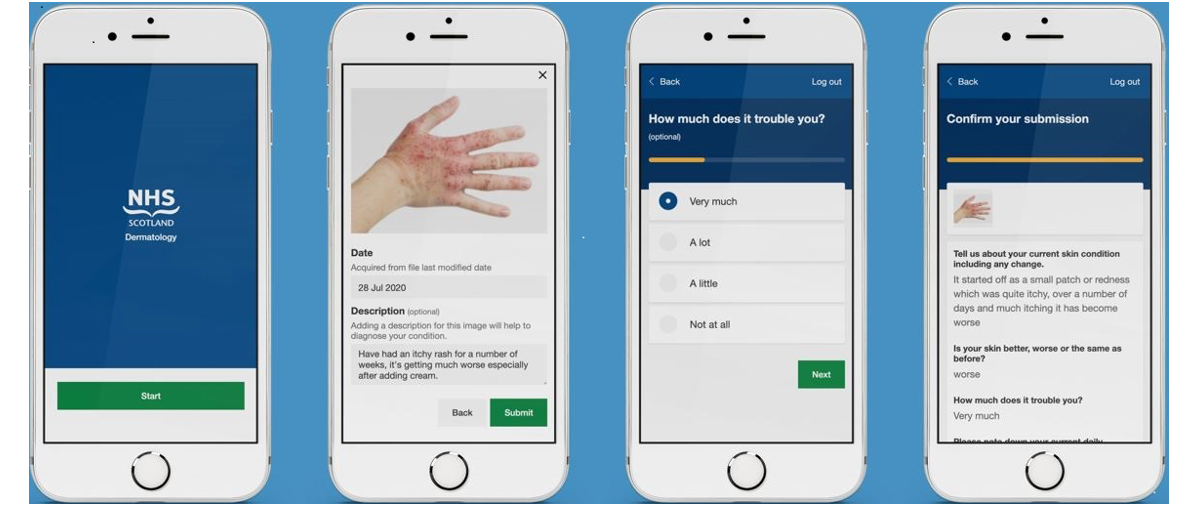
Patient interface. Patients are invited to register for the web-based platform via email. They can upload up to 4 images of their skin and are prompted to answer 6 questions about their presentation.

The real-time capture of data was undertaken as part of a cross-sectional evaluation of 405 digital assessments performed from March to June 2020 across 2 Scottish health boards (NHS Forth Valley and NHS Greater Glasgow and Clyde). Patients referred from primary care urgently with suspected skin cancer, in addition to urgent general dermatology referrals, were triaged for their suitability for this system and offered a digital assessment. Patients were invited to register and provide consent within the platform. They then had 5 days to upload 1 to 4 images and answer 6 questions about their presentation (Figure 1).

The application developed for this study provides bespoke clinician and patient interfaces. The clinicians’ individual dashboard allows them to view submissions from their assigned patients; if more information is needed, a direct request can be made to patients via the built-in messaging system. The clinician then sends a response with their diagnosis and management plan. A PDF summary is automatically sent to the primary care clinician, and a copy is stored on the electronic patient record.

Following each digital assessment, clinicians were asked to complete a standardized questionnaire, detailing the diagnosis, image quality, outcome, and time taken to complete the response (recorded by a stopwatch). The equivalent appointment length for a face-to-face consultation was stated by the clinician to permit comparison with conventional work patterns. Patient feedback was formally collated via a web-based survey of their user experience. This included specific questions on the ease of use, need for technical support, quality of care provided, travel time saved, and overall experience with the web-based platform.

### Ethics Approval

As the new system was introduced as part of service development (albeit in an accelerated way due to the pandemic), any evaluation of its acceptability and effectiveness would be considered a service evaluation or clinical audit. This means that the evaluation would not be considered NHS research and therefore had no need of either an NHS Research Ethics Committee review and opinion, or of NHS R&D approval from the participating Health Boards. The policy that service evaluations/audits do not require either NHS Research Ethics Committee review or NHS R&D approval dates back to the introduction of the Research Governance Framework (RGF) released in 2005. This was rolled over to the UK Policy Framework for Health and Social Care Research which superseded the RGF in October 2017 and was therefore applicable during the COVID-19 pandemic.

## Results

During the 11 weeks of the first national lockdown, 405 digital assessments were completed for 394 patients (Table 1). Of the 405 assessments, 297 (73.3%) were for new referrals, while 108 (26.7%) were return consultations. Patient ages ranged from 1 to 98 (mean 48) years, with 30.7% (121/394) aged older than 60 years, and parents of 12 children successfully completed digital assessments. Two-thirds of patients used a smart device, with the remainder using a PC or laptop (263/394). Further, 80% (324/405) of submitted images were considered of satisfactory quality by the assessing clinician, 41 patients under the government’s shielding program (identifying patients at the highest risk of COVID-19) received outpatient care from the safety of their home, and 218 consultations were carried out by a clinician working from home, highlighting the potential for digital dermatology assessments to provide a flexible alternative to traditional working patterns.

**Table 1:** Distribution of diagnoses (lesions vs inflammatory dermatoses).

Diagnoses			Distribution, n
**Lesions**			292
	Benign naevus	80	
	Actinic keratosis or Bowen disease	37	
	Seborrheic keratosis	33	
	Atypical naevus	29	
	Basal cell carcinoma	21	
	Solar lentigo	12	
	Vascular lesion	10	
	Squamous cell carcinoma or keratoacanthoma	7	
	Benign nail lesion	7	
	Viral wart	7	
	Dermatofibroma	4	
	Melanoma	3	
	Lentigo maligna	2	
	Other benign lesion^a^	26	
	Unknown diagnosis	10	
	Postbiopsy or photodynamic therapy review	4	
**Inflammatory dermatoses**			113
	Psoriasis	44	
	Eczema or seborrheic dermatitis	33	
	Acne or rosacea	9	
	Nodular prurigo or lichen simplex	4	
	Urticaria	3	
	Lupus	3	
	Vasculitis	2	
	Infective (tinea or impetigo)	2	
	Folliculitis	2	
	Postinflammatory hyperpigmentation	2	
	Indeterminate rash	2	
	Other inflammatory dermatoses^b^	7	

^a^Other benign lesions included Spitz naevus, clear cell acanthoma, glomus tumors, epidermoid cysts, sebaceous hyperplasia, and chondrodermatitis nodularis helicis.

^b^Other inflammatory dermatoses included panniculitis, lichen planus, pyoderma gangrenosum, necrobiosis lipoidica, dermatitis herpetiformis, pemphigus foliaceus, and telangiectasia macularis eruptiva perstans.

Of the 405 assessments, 292 (72.1%) concerned lesions, with the majority (241/292, 82.5%) referred for suspected cancer. Of these 292 lesions, 21 (7.2%) were booked for a web-based review, 139 (47.6%) were booked for a face-to-face review, 46 (15.8%) were directly booked for surgery, 81 (27.7%) resulted in discharge, and 5 (1.7%) were referred for photodynamic therapy. The remaining 113 (27.9%) assessments were for inflammatory dermatoses. Of these dermatoses, 45 (39.8%) were recommended for a web-based follow-up, 51 (45.1%) were recommended for a face-to-face review, 8 (7.1%) resulted in discharge, and 9 (8%) were referred for further investigations or treatments (Table 2). The majority of those needing a face-to-face review (137/190, 72.1%) were scheduled for a routine follow-up, although 27.9% (53/190) did require an urgent one-stop review for the confirmation of lesion diagnosis (including patients whose submitted images were unsatisfactory).

**Table 2:** Outcomes for digital assessments during lockdown.

		Outcomes, n (%)
**Overall outcomes (N=405)**		
	Further web-based review	66 (16.3)
	Face-to-face review	190 (46.9)
	Direct to biopsy	46 (11.4)
	Discharged	89 (22)
	Other treatments or investigations (phototherapy, patch testing, and PDT^a^)	14 (3.5)
**Outcomes for lesions (n=292)**		
	Further web-based review	21 (7.2)
	Face-to-face review	139 (47.6)
	Direct to biopsy	46 (15.8)
	Discharged	81 (27.7)
	Other treatments or investigations (PDT)	5 (1.7)
**Outcomes for inflammatory dermatoses (n=113)**		
	Further web-based review	45 (39.8)
	Face-to-face review	51 (45.1)
	Direct to biopsy	0 (0)
	Discharged	8 (7.1)
	Other treatments or investigations (phototherapy and patch testing)	9 (8)

^a^PDT: photodynamic therapy.

Additional information was acquired in 1 health board regarding histological diagnoses in 42 patients who were directly referred for biopsy or surgery following their assessment. This showed that 3 melanomas and 8 nonmelanoma skin cancers had been identified and treated.

Consultations in 1 health board were timed by using a stopwatch. The mean time for a total of 312 timed digital assessments was 10 minutes, whereas equivalent face-to-face consultations averaged 13 minutes. The time saving of digital consultations points to efficiency gains as well as the potential to increase available capacity and free up face-to-face appointments for those with complex care needs.

Feedback surveys indicated that satisfaction was high, with 82.1% (92/112) of respondents across both boards reporting ease of use. Further, 42% (47/112) reported that they would have normally needed to take time off work to attend a face-to-face appointment, while 21% (24/112) would have needed to travel for greater than 30 minutes to reach their hospital. Patient comments were generally positive, with many reporting that the reduced need for travel and time off work was a key benefit. Some patients reported technical difficulties, of which many have been subsequently improved after further development of the software. Newly referred patients were more likely to report that they would like more open dialogue with a clinician. Avoiding unnecessary hospital visits reduces the carbon footprint of the NHS and its contribution to the current climate emergency. During this evaluation, a total of 5758 km of patient travel was avoided through the use of digital assessments. This is equivalent to avoiding 719 kg of vehicle carbon dioxide emissions.

## Discussion

### Principal Findings

The COVID-19 pandemic has seen a paradigm shift in medical consultations, with telemedicine expanding rapidly to replace face-to-face interactions and reduce infection risk. A US institution reported a reduction in in-person visits to 1% of the prepandemic visit volume, with asynchronous consults accounting for 1 in 5 of all visits early in the pandemic [9]. Further, a questionnaire answered by dermatologists from 49 countries has confirmed the increased use and first-time adoption of teledermatology during the pandemic [10]. Improved patient access and staff productivity are also cited as reasons for continued use after pandemic lockdowns. We report the successful implementation of a digital assessment platform that has minimized face-to-face interactions, although we recognize that certain patients find digital assessment challenging, and others may prefer a real-time video or face-to-face consultation. Patients with established chronic dermatoses and those requiring a review of systemic therapies may be the most suited to digital assessment. We have been surprised by the utility of the platform in triaging and assessing skin lesions. Although this consultation platform demonstrates time savings per consultation, it did generate more review consultations. We recognize that patients requiring ongoing follow-up in dermatology would benefit from a mix of face-to-face and web-based consultations. The digital platform, which has now been evaluated and implemented in a third health board (NHS Grampian), is available for procurement by other health boards across the country.

Although we acknowledge that the pandemic necessitated travel restrictions, the system would ordinarily confer significant environmental benefits and improved convenience for patients. The greater adoption of digital consultations within high-footfall specialties, such as dermatology, could confer significant reductions in the carbon footprint of health care while also enabling patients to access flexible and convenient specialist assessments [11].

To date, over 3500 appointments have been completed, with more than 2500 occurring during mandated lockdown periods. This resulted in 150 hours of clinician time saved, representing a significant improvement in efficiency. The pandemic represents a paradigm shift in working patterns, and the digital solution reported herein, which is supported by dermatology patients and clinicians, is now supporting pandemic recovery. Interest in the platform has also been expressed by other specialties, including rheumatology, gastroenterology, and orthopedics.

### Conclusions

The novel store-and-forward asynchronous digital platform we describe offers an additional choice for providing outpatient care. The integration of the platform with the hospital booking system and patient record improved the efficiency and convenience of use. Digital consultations were typically shorter than conventional consultations, but around 50% (190/405, 46.9%) of patients did require a subsequent face-to-face review. The potential convenience for patients (eg, avoiding the need for travel and, beyond lockdown, avoiding the need to leave work or arrange childcare) was highly valued. Patients with a known diagnosis requiring follow-up may be the most suitable for the platform, but our experience indicates the potential utility of digital assessments for lesions and presentations of new dermatoses. An asynchronous digital platform complements phone, video, and conventional face-to-face consultations in offering choices in patient care, but it should only be offered within an integrated service.

## Abbreviations

NHS: National Health Service
